# Editorial: Unconventional protein secretion: From basic mechanisms to dysregulation in disease

**DOI:** 10.3389/fcell.2022.1088002

**Published:** 2022-11-30

**Authors:** Marioara Chiritoiu-Butnaru, Sarah E. Stewart, Min Zhang, Vivek Malhotra, Julien Villeneuve

**Affiliations:** ^1^ Department of Molecular and Cellular Biology, Institute of Biochemistry of the Romanian Academy, Bucharest, Romania; ^2^ Department of Biochemistry and Chemistry, La Trobe Institute for Molecular Science, La Trobe University Bundoora, Melbourne, VIC, Australia; ^3^ Tsinghua University, School of Pharmaceutical Sciences, Beijing, China; ^4^ Centre for Genomic Regulation (CRG), The Barcelona Institute for Science and Technology, Barcelona, Spain; ^5^ Universitat Pompeu Fabra (UPF), Barcelona, Spain; ^6^ ICREA, Barcelona, Spain; ^7^ Institute of Functional Genomics, University of Montpellier, CNRS, INSERM, Montpellier, France

**Keywords:** unconventional protein secretion (UPS), membrane trafficking, cell adaptation, disease, cell compartmentalization

In eukaryotes, the classical view is that secreted proteins involved in intercellular communication are exported from cells through a highly conserved pathway generally termed the conventional secretory pathway, first postulated by Palade and co-workers (Palade, 1975). Over the past 5 decades, this pathway has been extensively studied, leading to an extensive characterization of key players involved in cargo recognition, packaging, sorting and transport. Briefly, proteins released by cells through the conventional secretory pathway, such as antibodies, collagens, mucins, cytokines, hormones, and neurotransmitters, contain a signal sequence that directs their translocation into the Endoplasmic Reticulum (ER) where they are packaged and sorted by the use of COPII coats and TANGO1 (transport and Golgi organization 1) to reach the Golgi apparatus (Raote et al., 2021). Then, cargo proteins are resorted at the Golgi apparatus and delivered by vesicular transport to their respective destinations within the cell and to the cell’s exterior. Although challenging questions remain unanswered, understanding these processes has revealed their fundamental role in maintaining the specificity and communication of different organelles, in supporting cell-cell communication, and has highlighted how defects in this secretory route are connected to human diseases.

However, new findings have recently emphasized the critical role of alternative secretory pathways for the export of an increasing number of proteins lacking signal sequence (or leaderless proteins). These new routes, collectively designated as unconventional protein secretion (UPS), have emerged as essential for maintaining cellular homeostasis and intercellular communication. Proposed pathways involve sequential events that take place at the plasma membrane (PM) where leaderless proteins can be directly translocated across the lipid bilayer through protein channels, or transferred to adjacent cells through microvesicles or tunnelling nanotubes. Other underlying mechanisms reflect the plasticity and dynamic properties of intracellular compartments that can be remodeled, rerouted or created *de novo* in response to intrinsic demands or external signals for secretion of signal sequence lacking proteins (Filaquier et al., 2022) (Figure 1).

**FIGURE 1:**
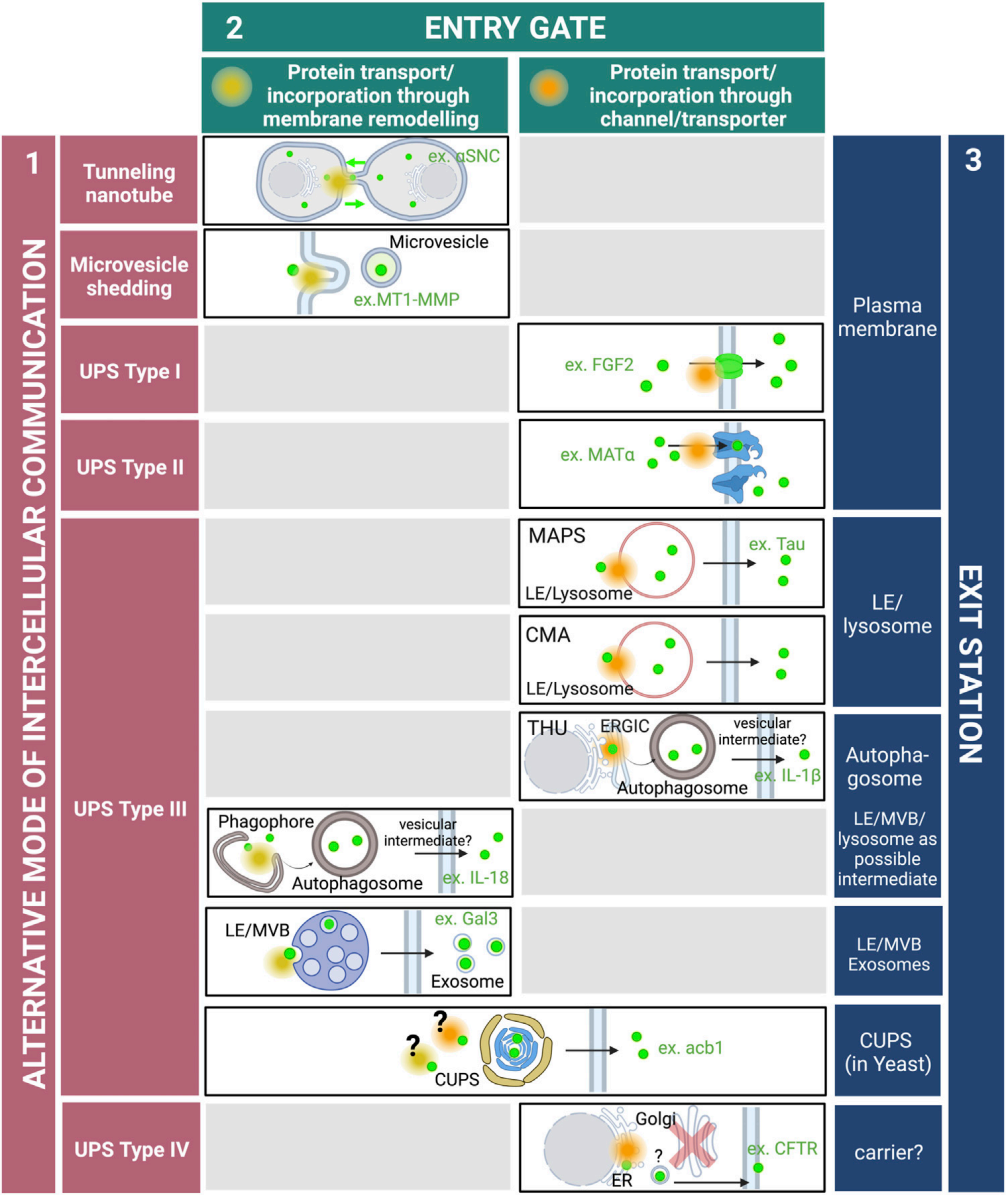
Roads and hubs of unconventional protein secretion. In addition to the conventional ER-Golgi secretory pathway, cells are endowed with additional routes for the export of cytoplasmic proteins, and multiple modes of intercellular communications have been characterized (purple). They include tunnelling nanotubes formed by membranous protrusions that emerge from the PM and connect adjacent cells. Tunnelling nanotubes allow the direct transfer of cytoplasmic proteins as well as whole organelles such as lysosomes. Outward budding of the PM produce microvesicles that are then released into the extracellular space. In types I and II UPS, cargo proteins are directly translocated across the PM by pore formation or ABC transporters, respectively. In type III UPS, cargo proteins are incorporated into intracellular compartments and transported through single or successive membrane intermediates that fuse with the PM. In type IV UPS or Golgi-bypass, integral membrane proteins are inserted into the ER and then reach the PM independently of the Golgi apparatus. Along these alternative secretory routes, cargo proteins are transported or incorporated within membrane compartments either by protein channels or by membrane remodeling defining distinct entry gates at the PM or along intracellular trafficking pathways (green). Cargo proteins can also be gathered into particular membrane intermediates prior to release into the extracellular space, defining distinct exit stations (blue). Abbreviations–αSNC: alpha-synuclein; acb1: acyl-CoA-binding protein; CFTR: cystic fibrosis transmembrane conductance regulator; CUPS: Compartment for unconventional protein secretion; CMA: Chaperone-mediated autophagy; ER: Endoplasmic reticulum; ERGIC: ER-Golgi intermediate compartment; FGF2: Fibroblast growth factor 2; Gal3: Galectin-3; IL: Interleukin; LE: Late endosome; MAPS: Misfolded-associated protein secretion; MT1-MMP: Membrane-type 1 matrix metalloproteinase; MVB: Multivesicular body; THU: TMED10-channeled UPS; UPS: Unconventional protein secretion. The figure was generating using BioRender.

This new paradigm led us to revisit the current framework of mechanisms that support protein trafficking and secretion. This Research Topic of 18 articles explores the general topic of UPS by covering basic mechanisms, their dysregulation in pathophysiological conditions, and the potential use of UPS for biomedical applications.

## UPS mediated by vesicular intermediates (type III and IV UPS)

UPS has been identified in all eukaryotes including plants, yeast, flies and mammals to export a wide range of protein families such as cytokines, lipid chaperones, hydrolytic enzymes, or toxic aggregate-prone proteins, among others. This raises the central question of the evolutionary significance of UPS and the selective advantages gained by acquiring these alternative secretory routes. To address this issue, the comprehension of UPS mechanisms for multiple cargo proteins in various organisms is required. Maricchiolo et al. describe mammalian and plant UPS pathways, pointing similarities and differences, and propose revising UPS plant classification to converge on a single classification system based on features defined in mammalian UPS. While UPS relies on a striking diversity of mechanisms in both plants and animals, common processes are highlighted, as illustrated by intracellular compartments with equivalent functions, such as the vacuole in plants and lysosomes in animals that can be diverted into secretory organelles, thus representing important sorting stations for UPS. However, the involvement of additional vesicular intermediates that may derive from autophagosomes, multivesicular bodies (MVBs) or, as shown in yeast, from a transient and hybrid compartment formed by Golgi and endosome membranes called CUPS, have also been reported (Rabouille, 2017). Lee et al. describe recent advances in a specific UPS pathway called MAPS (Misfolded associated protein secretion) that requires the mobilization of consecutive vesicle carriers along the endo-lysosomal system. MAPS is used by cells as an additional protein quality control (PQC) mechanism in the context of proteotoxic stress to promote clearance of misfolded proteins. This UPS pathway is controlled by the coordinated action of the ER associated deubiquitylase USP19, the membrane-associated chaperone HSC70 and its co-chaperone DNAJC5/CSPα, the latter being able to couple MAPS with the endosomal microautophagy. Noh et al. focus on autophagy-related pathways and describe in detail how autophagy machineries can alter UPS. While autophagosomes could represent a major entry gate for many UPS cargo proteins, there is no evidence that autophagosomes directly fuse with the PM. Instead, autophagosomes might first fuse with other vesicular intermediates, such as late endosomes, MVBs or lysosomes, which then release their content into the extracellular space after exocytosis. Vats and Galli describe the role of the machinery involved in fusion events between distinct intracellular compartments and the PM. Specifically, they highlight the role of the vesicular SNARE protein VAMP7, which has been reported to be involved in the exosome-, lysosome- and autophagy-mediated secretion.

While UPS is mainly involved in the export of proteins lacking a signal sequence, mechanisms allowing the transport of proteins from the ER to the cell surface independently of the Golgi apparatus have also been indexed as UPS pathway. This is illustrated by the study of Dimou et al. which suggests the existence of distinct COPII carriers involved in the transport from the ER to the PM of the proton-pump ATPase PmaA and the PalI pH sensing component, in the filamentous fungus *Aspergillus nidulans*.

## UPS mediated by direct translocation across the plasma membrane (type I UPS)

In addition to pathways mediated by vesicular intermediates, UPS is also achieved by pore-mediated translocation across the PM. This direct mode of secretion has been extensively studied in the case of Fibroblast growth factor 2 (FGF2), for which detailed mechanistic insights have been provided (Sparn et al., 2022). Briefly, FGF2 secretion is mediated by sequential interactions of FGF2 with Na,K-ATPase, tec kinase and phosphoinositide in the inner leaflet of the PM. These interactions trigger FGF2 oligomerization, leading to the formation of membrane-spanning FGF2 oligomers, recognized as a self-sustained translocation channel. Directional transport of FGF2 is then ensured by the interaction of FGF2 with heparan sulfate proteoglycans located on the outer leaflet of the PM. Here, Lolicato and Nickel discuss recent findings suggesting that this sequence of events occurs in specialized, liquid-ordered nanodomains of the PM enriched in cholesterol and phosphatidylinositol 4,5-bisphosphate (PI(4,5)P_2_). Other examples of UPS cargo proteins directly translocated across the PM include Tau, HIV-Tat and Engrailed 2 homeoprotein. Joliot and Prochiantz comprehensively summarize the mechanisms involved in the UPS of homeoproteins that also require their physical interactions with PI(4,5)P_2_ and proteoglycans, and focus on their physio-pathological functions once secreted and internalized by adjacent cells.

The mechanisms involved in the secretion of IL-1 family cytokines are also of major interest and perfectly illustrate how UPS cargo proteins can be directed to multiples UPS pathways (Semino et al., 2018). While several studies have demonstrated that IL-1 family members can be secreted after being incorporated into intracellular compartments, including autophagosomes or lysosomes (Pallotta and Nickel, 2020), compelling evidence have recently highlighted that, during acute inflammation, IL-1β is translocated across the PM through a channel formed after oligomerization of inflammasome-activated Gasdermin D (Evavold et al., 2018). In this context, Evavold and Kagan discuss appealing emerging ideas whereby host metabolic state dictates alternative or complementary pathways for IL-1β secretion.

## Extracellular vesicles (EVs) and tunnelling nanotubes (TNTs)

UPS also includes secretory routes by which cargo proteins are released from cells enclosed in EVs. Meldolesi describes the general properties of the two main types of EVs, i.e. exosomes and microvesicles, and the mechanisms underlying their biogenesis. Briefly, exosomes derive from intraluminal vesicles (ILVs) formed within the endocytic pathway by inward budding of late endosomes. This generates MVBs that can then fuse with the PM, whereas microvesicles derive from outward budding of the PM. Although EVs differ in many features including size and molecular composition, the biogenesis of exosomes and microvesicles requires the endosomal sorting complex required for transport (ESCRT) machinery. Thuault et al. summarize the current knowledge of the molecular mechanisms involved in the loading of matrix metalloproteinases (MMPs) into both exosomes and microvesicles, focusing particularly on MT1-MMP, a membrane-associated MMP contributing to cell invasive behavior. They provide a detailed description of the underlying trafficking events that combine cycles of endocytosis, recycling and exocytosis. Bänfer et al. provide new insight in the loading of E-cadherin into ILVs, that depends on the ESCRT-I component Tsg101. Farley et al. summarize the state of knowledge on plant EVs, and present exciting perspectives on their potential use as drug delivery tools.

Finally, to complete the list of alternative modes of intercellular communication, Turos-Korgul et al. describe how membranous protrusions emerging from the PM to connect adjacent cells and called tunnelling nanotubes (TNTs) are established, and their role in the progression of a wide range of diseases.

## UPS in disease

Several review in this Research Topic, as described above, have extended their discussion to the role of UPS in several disorders. In addition, Iglesia et al. provide a comprehensive overview of UPS involvement in brain tumor maintenance, and Pilliod et al. present new findings on the UPS of Tau, whose release from neurons may represent a critical step in Alzheimer’s disease progression.

Thus, although further studies are needed to delineate the mechanisms and factors involved in the different UPS pathways, we now appreciate their fundamental role in cell biology and how their dysregulation is associated with diseases. Therefore, research on UPS will not only highlight processes conserved across many species, but also open new perspectives for the development of innovative therapeutic strategies. A promising direction will also be the development of new biotechnological applications, as illustrated by Philipp et al. who explore in a fungal model the use of UPS cargo proteins as a carrier for the production and export of heterologous proteins, including synthetic nanobodies directed against the SARS-CoV2 virus.

## Concluding remarks

In conclusion, while initially perceived as an enigma by the scientific community studying protein trafficking and secretion processes, the pioneer studies that revealed the lack of a signal peptide in the IL-1β sequence, and the release of this secreted cytokine through an ER-Golgi-independent pathway (Auron et al., 1984; Rubartelli et al., 1990), have laid the foundation for an entirely new field of research. The field is now maturing, suggesting even more questions and challenges in the coming years. For example, how are UPS pathways integrated into the adaptive stress response to meet cellular needs, and what is the functional relationship between conventional and unconventional secretory pathways? The role of GRASP proteins could be of major importance in this context, given their function in Golgi organization and their relocation to distinct organelles involved in UPS during particular stress conditions (Cruz-Garcia et al., 2014; Zhang et al., 2019; Nüchel et al., 2021). The principles of selection and recognition of cargo proteins for UPS also warrant further exploration? Specific domains or amino acids have been recognized for a few UPS cargo proteins, but they remain elusive in most cases. Cargo selection for UPS likely relies on a combination of specific and complementary sequences, as illustrated by Biswal et al. Another challenge will be to obtain a comprehensive list of cargo proteins that are actively and selectively secreted by UPS. Poschmann et al. examine how recent advances in quantitative secretomics combined with pharmacological perturbation strategies (pharmocosecretomics) could achieve this critical objective.

Overall, these contributions are representative of the complexity and diversity of the mechanisms underlying UPS, which intersect with key processes within the cell, including protein sorting, membrane trafficking, and organelle dynamics. Undoubtedly, the knowledge gained about these fundamental principles of cell biology, combined with the prodigious technological advances made in recent years, guarantees exciting new discoveries about UPS in the near future.
